# Phase II trial of eribulin mesylate as a first- or second-line treatment for locally advanced or metastatic breast cancer: a multicenter, single-arm trial

**DOI:** 10.1186/s12885-018-4628-7

**Published:** 2018-06-28

**Authors:** Tetsu Hayashida, Hiromitsu Jinno, Katsuaki Mori, Hiroki Sato, Akira Matsui, Takashi Sakurai, Hiroaki Hattori, Shin Takayama, Masahiro Wada, Maiko Takahashi, Hirohito Seki, Tomoko Seki, Aiko Nagayama, Akiko Matsumoto, Yuko Kitagawa

**Affiliations:** 1 Department of Surgery, Keio University School of Medicine, 35 Shinanomachi., Shinjuku, Tokyo, 160-8582 Japan; 2 Department of Surgery, Teikyo University School of Medicine, Tokyo, Japan; 3 Department of Surgery, Hino Municipal Hospital, Tokyo, Japan; 4 Department of Surgery, Mito Red Cross Hospital, Ibaraki, Japan; 5 Department of Surgery, National Hospital Organization Tokyo Medical Center, Tokyo, Japan; 6 Division of Surgery, JCHO Saitama Medical Center, Saitama, Japan; 7 Department of Surgery, Federation of National Public Service Personnel Mutual Aid Associations, Tachikawa Hospital, Tokyo, Japan; 8 Department of surgery, Tokyo Dental College Ichikawa General Hospital, Tokyo, Japan; 9 Department of Surgery, Sanokousei general hospital, Tochigi, Japan

**Keywords:** Breast cancer, Eribulin, Phase II trial

## Abstract

**Background:**

Eribulin mesylate is currently indicated as a sequential monotherapy to be administered after two chemotherapeutic regimens, including anthracycline and taxane treatments, for treatment of metastatic breast cancer. This open-label, multicenter phase II study was designed to evaluate the efficacy and safety of eribulin as a first- or second-line treatment for patients with metastatic breast cancer.

**Methods:**

The primary objective was to determine the overall response rate. Secondary objectives were to evaluate progression-free survival and the safety profile. Patients were scheduled to receive eribulin mesylate 1.4 mg/m^2^ intravenously on days 1 and 8 of a 21-day cycle. Patients received the study treatment unless disease progression, unacceptable toxicity, or a request to discontinue from the patient and/or investigator eventuated.

**Results:**

Between December 2012 and September 2015, 32 patients with metastatic breast cancer were enrolled at 10 participating clinical institutions in Japan, and toxicity and response rates were evaluated. The overall response rate was 43.8% (95% confidence interval [CI] 26.5–61.0). The clinical benefit and tumor control rates were 56.3% (95% CI 39.0–73.5) and 78.1% (95% CI 63.8–92.5), respectively. Median progression-free survival was 8.3 months (95% CI 7.1–9.4). A subgroup analysis did not identify any factors affecting the efficacy of eribulin. The most common adverse events were neutropenia (71.9%), alopecia (68.7%), and peripheral neuropathy (46.9%). As a first- or second-line therapy, eribulin showed sufficient efficacy for metastatic breast cancer compared with taxane and capecitabine treatment in previous clinical trials. The safety profile of eribulin was acceptable.

**Conclusions:**

Eribulin may be another option for first-line chemotherapeutic regimens for metastatic breast cancer.

**Trial registrations:**

This trial was retrospectively registered at the University Hospital Medical Information Network (UMIN) Clinical Trial Registry (ID number: UMIN000010334).

Date of trial registration: April 1st, 2013.

## Background

Most cases of metastatic breast cancer are not curable and the strategy for treatment aims to extend life, suspend cancer progression, remove cancer-related symptoms, and improve quality of life. [1] Patients may derive sustained benefits from the administration of anthracyclines and taxanes, which are the standard chemotherapeutics for metastatic breast cancer [2]; however, ongoing research efforts aim to increase the number of available agents with more efficacy and less toxicity to improve treatment strategies.

Eribulin mesylate (eribulin) is a non-taxane, microtubule dynamics inhibitor belonging to the halichondrin class of antineoplastic agents. The growth phase of microtubules is effectively suppressed by eribulin without affecting the shortening phase, and eribulin isolates tubulin into non-productive aggregates. [3] In a global phase III trial, a survival benefit was confirmed in women with heavily pretreated advanced breast cancer assigned to eribulin versus a control arm of patients receiving the physician’s choice of treatment (hazard ratio (HR) 0.81, 95% confidence interval [CI], 0.66–0.99; *P* = 0.041). [4] Eribulin also demonstrated a positive survival benefit compared with capecitabine in another phase III trial; however, this improvement did not meet the set criteria for statistical significance. [5] Moreover, a pooled analysis of the above-mentioned phase III studies demonstrated that eribulin-treated patients had a significantly extended overall survival (OS). [6]

Based on results from these studies, in the United States eribulin is currently indicated in metastatic breast cancer as a sequential monotherapy to be administered after two chemotherapeutic regimens, including anthracycline and taxane treatments. In Japan, however, eribulin has been approved for use for patients with metastatic breast cancer regardless of the number of pretreatment chemotherapeutic regimens.

The American Society of Clinical Oncology clinical practice guidelines suggest that no clear evidence exists for the superiority of one specific drug or regimen for first- and second-line treatment of patients with metastatic breast cancer. The guideline also recommends that previous therapy and differential toxicity should be considered for treatment selection, although anthracycline and taxanes have the strongest evidence for efficacy. [7] Therefore, eribulin, which has been proven effective and safe in heavily pretreated patients, is a possible candidate as an upfront agent for metastatic breast cancer. In this context, we conducted this single-arm, multicenter phase II trial to investigate the efficacy and safety of eribulin for first- and second-line treatment, which may provide another option for upfront chemotherapeutic regimens for patients with metastatic breast cancer.

## Methods

### Study design

This open-label, multicenter phase II study was designed to evaluate the efficacy and safety of eribulin as a first- or second-line treatment for patients with metastatic breast cancer. The primary objective was to determine the overall response rate (ORR). Secondary objectives were to evaluate progression-free survival (PFS), the clinical benefit rate (CBR), the tumor control rate, the objective response rates for patient subgroups, and the safety profile of eribulin. CBR was defined as the proportion of patients with a complete response (CR) or a partial response (PR), or with stable disease at 6 months. The tumor control rate was defined as the proportion of patients who achieved a CR or PR, or with stable disease. Subgroup analyses were performed based on receptor status, metastatic site, and dose reduction during treatment.

### Eligibility criteria

Women who met the following criteria were eligible for inclusion: histologically or cytologically confirmed recurrent or metastatic adenocarcinoma of the breast with at least 1 measurable lesion, according to the Response Evaluation Criteria in Solid Tumors (RECIST) version 1.1.; an Eastern Cooperative Oncology Group (ECOG) performance status score of 0, 1, or 2; a life expectancy of more than 12 weeks; up to one prior chemotherapy regimen for advanced and/or metastatic disease; a normal electrocardiogram; and laboratory cut-off values, as follows: neutrophil count ≥1.5 × 10^9^/L, platelet count ≥100 × 10^9^/L, hemoglobin level ≥ 9.0 g/dL, serum bilirubin level < 2.0 × the upper limit of the normal level, and aspartate aminotransferase (AST), alanine aminotransferase (ALT), and alkaline phosphatase levels < 2.5 × the upper limit of the normal level, and a serum creatinine level < 1.5 mg/dL. Patients were excluded if they had prior eribulin treatment or had been diagnosed with a serious concomitant illness such as uncontrolled diabetes, severe cardiovascular disease, interstitial pneumonia, lung fibrosis, or active concomitant malignancy. Pregnant or lactating women were also excluded.

### Treatment plan

Patients were scheduled to receive eribulin mesylate 1.4 mg/m^2^ intravenously on days 1 and 8 in each 21-day cycle. Patients received study treatment unless disease progression, unacceptable toxicity, or a request to discontinue from the patient and/or the investigator eventuated. Toxicities were evaluated according to the National Cancer Institute Common Terminology Criteria for Adverse Events (CTCAE version 4) throughout treatment with eribulin. Treatment could be postponed for a maximum of 3 weeks for severe toxicity. Dose reductions of eribulin from 1.4 mg/m^2^ to 1.1 mg/m^2^ and from 1.1 mg/m^2^ to 0.7 mg/m^2^ were permitted in cases of febrile neutropenia and grade 3 or 4 non-hematological toxicities, respectively. G-CSF was appropriately used according to the guideline made by Japan Society of Clinical Oncology.

### Response evaluation

Tumor response was determined according to RECIST version 1.1 and had to be confirmed after every 3 cycles using spiral computed tomography or magnetic resonance imaging. When a symptom suggesting bone metastasis was observed, bone scintigraphy was performed. Patients who discontinued treatment due to adverse events (AEs) prior to the first evaluation were categorized as not evaluated (NE). After first observation of a CR or a PR, tumor response was confirmed at a second assessment 4 weeks later. Time to progression was determined as the interval from the start of treatment to the date of the documented tumor progression, or the date of death from any cause if the patient died prior to documentation of disease progression.

### Statistical analyses

Efficacy analyses were performed on the intention-to-treat population. The primary objective of this study was to show adequate activity of eribulin treatment measured using objective response rates. For an appropriate sample size calculation and using a two-sample t-test to test the null hypothesis of a true response rate of 20% against the alternative hypothesis of a true response rate of almost 40%, 32 assessable patients had to be included (α = 0.05; β = 0.8). Therefore, we set a final sample size at 35 patients. Tumor assessments were obtained from an investigator radiology review.

## Results

### Patient characteristics

Between December 2012 and September 2015, 35 patients with metastatic breast cancer were assigned to our clinical trial at 10 participating clinical institutions in Japan. Two patients did not meet the criteria and one patient withdrew consent prior to treatment; therefore, 32 patients were enrolled and evaluated for toxicity and response rates. Baseline patient demographics and disease characteristics are shown in Table 1. Twenty-two (68.8%) patients received eribulin as a first-line chemotherapy for advanced disease. Five patients (15.6%) had received taxane and 5 patients (15.6%) had received oral 5-FU (capecitabine or S-1) prior to eribulin treatment.

**Table 1:** Patient demographics and disease characteristics

	No. of patients	%
Age, years		
Median	66	
Range	39–82	
ECOG performance status		
0	25	78.1
1	5	15.6
2	2	6.3
(Neo-)adjuvant chemotherapy	22	68.7
Anthracycline	7	21.9
Taxane	2	6.3
Anthracycline + taxan	11	34.4
Oral 5FU	1	3.1
CMF	1	3.1
Adjuvant endocrine therapy	14	43.7
Prior endocrine therapy for advanced disease	15	46.9
No. of prior chemotherapy regimens for advanced disease		
0	22	68.8
1	10	31.3
Taxan	5	15.6
Oral 5FU (capecitabine or S-1)	5	15.6
ER-postive	19	59.4
PgR-positive	18	56.3
HER2-positive	1	3.1
Triple negatve	11	33.3
Metastatic site		
Liver	14	43.8
Lung	14	43.8
Brain	0	0
Bone	13	40.6
Skin	8	25
Other	14	43.8
No of organs involved		
1	9	28.1
2	13	40.6
3	5	15.6
4	5	15.6
Site of disease		
Viceral	23	71.9

Abbreviations: *ECOG* Eastrern cooperative oncology group, *ER* estrogen receptor, *PgR* progesterone receptor, *HER*2 human epidermal growth factor receptor 2

### Efficacy

**Fig. 1:**
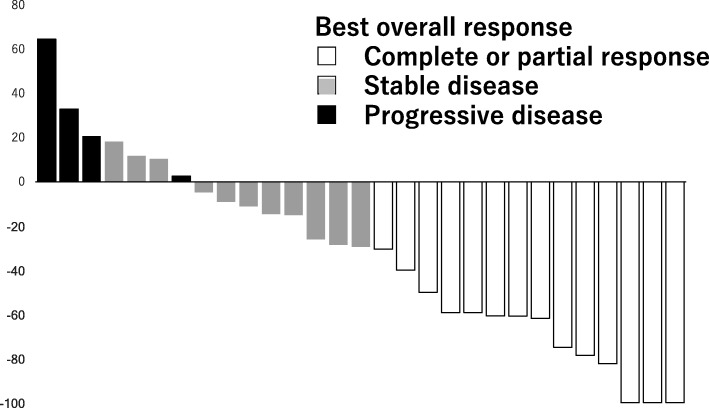
Waterfall graphs of percentage change in the total sum of target lesion diameters from baseline to postbaseline nadir (RECIST v1.1)

**Fig. 2:**
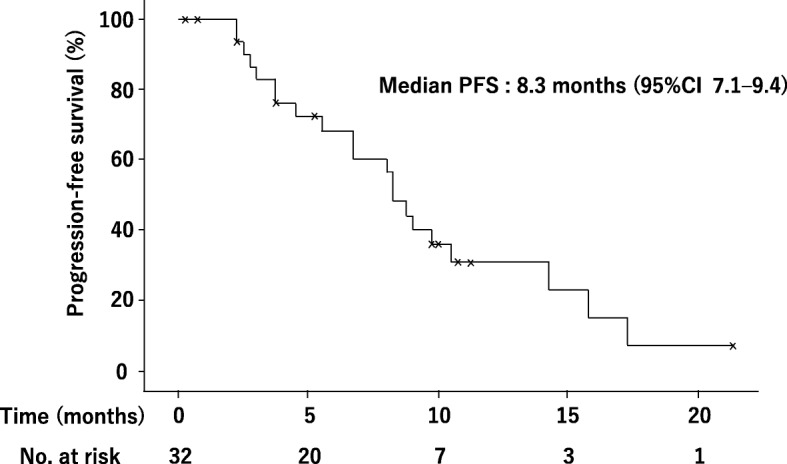
Kaplan-Meier plot of progression-free survival

At the time of this analysis, a total of 32 patients were assessable for efficacy. The ORR was 43.8% (95% CI 26.5–61.0) (Table 2). CBR and tumor control rates were achieved in 56.3% (95% CI 39.0–73.5) and 78.1% (95% CI 63.8–92.5) of patients, respectively. The maximum change in tumor size for each patient is shown in Fig. 1. The median PFS was 8.3 months (95% CI 7.1–9.4) (Fig. 2). Subgroup analysis did not identify any factor affecting the efficacy of eribulin with statistical significance (Table 3).

**Table 2:** Efficacy outcomes

	No. of patients	%	95%CI
All assessable patients	32		
CR	3	9.4	
PR	11	34.4	
SD	11	34.4	
PD	4	12.5	
NE	3	9.4	
Overall response (CR + PR)	14	43.8	(26.5–61.0)
Clinical benefit rate (CR + PR + SD≧6 months)	18	56.3	(39.0–73.5)
Tumor control rate (CR + PR + SD)	25	78.1	(63.8–92.5)

**Table 3:** Objective response rates for over all population and subgroups of patients

	N	Overall response rate (%)	Clinical benefit rate (%)	Median PFS (95%CI), months
Overall	32	43.8	56.3	8.3 (7.1–9.4)
Hormone receptor status				
HR(−)	12	33.3	41.7	5.5 (0–11.5)
HR(+)	20	50	65	8.8 (6.46–11.0)
No. of prior chemotherapy				
0	22	50	54.5	8.8 (7.4–10.1)
1	10	30	70	8.3 (3.7–12.8)
Metastatic site				
Visceral	23	43.5	60.9	8.3 (7.2–9.3)
Non-visceral	9	44.4	44.4	9.0 (4.2–13.8)
Dose reduction during treatment				
No reduction	18	33.3	50	8.8 (3.9–13.6)
Reduction	14	57.1	64.3	8.3 (7.8–8.7)
Hormone receptor status				
Triple negative	11	36.4	36.4	5.5 (1.3–9.7)
Other	21	47.6	71.4	8.8 (7.0–10.5)

### Safety

All patients experienced a treatment-related adverse event (AE). The most common AEs were neutropenia (71.9%), alopecia (68.7%), and peripheral neuropathy (46.9%) (Table 4). Twelve patients experienced a SAE: 7 grade 4 neutropenia, 1 febrile neutropenia, 4 grade 3 fatigue.

**Table 4:** Treatment-related adverse events

	Any grade	Grade 3	Grade 4
Hematologic			
Neutropenia	23 (71.9)	6 (18.8)	7 (21.9)
Anemia	7 (21.9)	1 (3.1)	0
Thrombopenia	5 (15.6)	2 (6.3)	0
Febrile Neutropenia	1 (3.1)	1 (3.1)	0
Nonhematologic			
Fatigue	16 (50.0)	4 (12.5)	0
Alopecia	22 (68.7)	N/A	
Peripheral neuropathy	15 (46.9)	4 (12.5)	0
Arthralgia	4 (12.6)	0	0
Constipation	7 (21.9)	1 (3.1)	0
Diarrhea	2 (6.2)	0	0
Nausea	8 (25.0)	0	0
Vomitting	1 (3.1)	0	0
Stomatitis	9 (28.1)	2 (6.3)	0

Treatment for AEs led to dose reductions for 14 patients (43.8%), and these dose reductions were most commonly due to neutropenia and peripheral neuropathy. Two patients (6.2%) discontinued eribulin treatment prior to the first evaluation. One patient discontinued eribulin after 2 cycles of treatment due to grade 3 fatigue. Another patient was discontinued after a cycle also due to grade 3 fatigue. A patient died because of aggressive disease progression after 2 cycles of treatment.

## Discussion

This single-arm, multicenter phase II study, assessing first- and second-line treatment with eribulin monotherapy for advanced breast cancer, found a 43.8% ORR and a median PFS of 8.3 months. These outcomes are not inferior to several randomized control studies using paclitaxel monotherapy for advanced breast cancer in the control arm, for which the reported range of ORR is between 21.2 and 26.2%. [3, 8] In some situations, oral 5-FU agents may be selected as upfront treatment for advanced breast cancer. First-line capecitabine monotherapy has an ORR of 22% and a PFS of 6 months, [9] which is consistent with the findings of the current trial. Miller et al. reported that patients (*n* = 346) who received 90 mg/m^2^ of paclitaxel on days 1, 8, and 15 of every 28-day cycle as first-line chemotherapy had a 21.2% ORR and a PFS of 5.9 months. [8] In this study, adding bevacizumab to paclitaxel improved the ORR to 36.9% and the PFS to 11.8 months; however, OS was similar to paclitaxel monotherapy. On the other hand, a pooled analysis of the EMBRACE and the 301 trials confirmed that eribulin-treated patients had a significantly extended OS. [6]

Recent preclinical studies have provided important information on how eribulin prolongs OS. Yoshida et al. reported that treatment of triple negative breast cancer cells with eribulin led to morphological and molecular changes consistent with transition of a mesenchymal to an epithelial phenotype through inhibition of SMAD2 and SMAD3 phosphorylation. [10] Several studies have also suggested that eribulin changes microenvironmental vascular networks around tumors. Eribulin, but not paclitaxel, showed strong efficacy as an antivascular agent that affected pericyte-driven angiogenesis. [11] Another study revealed that eribulin-induced remodeling of tumor vasculature leading to a more functional microenvironment that may reduce the aggressiveness of tumors. [11] Eribulin treatment also increased tumor oxygen saturation and decreased the plasma concentration of TGF-beta1 leading to a favorable anti-angiogenic effect. [12] These capabilities of eribulin, demonstrated through preclinical studies to reverse the aggressive characteristics related to both cellular phenotype and microenvironment, may be contributing to its clinical benefits. Therefore, the strategy of upfront treatment with eribulin may be able to reduce the aggressiveness of tumors, which contributes to the efficacy of later management with another form of chemotherapy, and to prolonging patient survival.

McIntyre et al. and Takashima et al. each conducted and reported phase II studies to investigate the efficacy and safety of first-line eribulin treatment for metastatic breast cancer, and reported ORRs of 29 and 54.3%, respectively. [13, 14] Despite including second-line treatment in our study, the ORR was 43.8% and this result is similar to previous first-line trials. Moreover, the PFS in this study was superior to previous studies; however, the difference may be due to bias in relation to the study design. Radiographic evaluation was undertaken after every 3 cycles of eribulin treatment in this study, but an evaluation was conducted after every 2 cycles of eribulin treatment in the first-line trials, suggesting that the PFS in this study might be overestimated. However, in an actual clinical situation, assessments using computed tomography and bone scintigraphy might not be conducted every 6 weeks. Therefore, it is likely that our data may be relatively close to a real-world situation.

The safety profile of eribulin in upfront treatment was similar to that identified in previous studies. [4, 5, 13, 14] The most frequent non-hematologic AEs of any grade were fatigue (50%), alopecia (68.7%), and peripheral neuropathy (46.9%). Neutropenia was the most frequent grade 3 (18.8%) or 4 (21.9%) AE. Febrile neutropenia was reported only in one patient. Dose reductions were most commonly due to neutropenia and peripheral neuropathy; however, the efficacy of eribulin did not change in the subgroup analysis. Therefore, patients may have some clinical benefit from continuous treatment with eribulin regardless of dose intensity.

## Conclusion

Overall, as a first- or second-line therapy, eribulin showed comparable efficacy for metastatic breast cancer in comparison with a single treatment of taxane and oral 5-FU as a first-line therapy as shown in previous clinical trials. Eribulin also demonstrated acceptable safety and tolerability. These results suggest that eribulin may have clinical benefits as an upfront chemotherapeutic regimen for metastatic breast cancer patients.

## Abbreviations

AE: Adverse event
ALT: Alanine aminotransferase
AST: Aspartate aminotransferase
CR: Complete response
CTCAE: National Cancer Institute Common Terminology Criteria for Adverse Events
ECOG: Eastern Cooperative Oncology Group
ORR: Overall response rate
OS: Overall survival
PFS: Progression-free survival
PR: Partial response
RECIST: Response Evaluation Criteria in Solid Tumors
